# HMG-CoA Reductase Inhibition Promotes Neurological Recovery, Peri-Lesional Tissue Remodeling, and Contralesional Pyramidal Tract Plasticity after Focal Cerebral Ischemia

**DOI:** 10.3389/fncel.2014.00422

**Published:** 2014-12-11

**Authors:** Ertugrul Kilic, Raluca Reitmeir, Ülkan Kilic, Ahmet Burak Caglayan, Mustafa Caglar Beker, Taha Kelestemur, Muhsine Sinem Ethemoglu, Gurkan Ozturk, Dirk M. Hermann

**Affiliations:** ^1^Department of Physiology, Istanbul Medipol University, Istanbul, Turkey; ^2^Department of Neurology, University Hospital, Essen, Germany; ^3^Department of Medical Biology, Istanbul Medipol University, Istanbul, Turkey

**Keywords:** middle cerebral artery occlusion, neurological recovery, neuronal plasticity, restorative therapy, statin, tract tracing

## Abstract

3-Hydroxy-3-methylglutaryl-coenzyme A (HMG-CoA) reductase inhibitors are widely used for secondary stroke prevention. Besides their lipid-lowering activity, pleiotropic effects on neuronal survival, angiogenesis, and neurogenesis have been described. In view of these observations, we were interested whether HMG-CoA reductase inhibition in the post-acute stroke phase promotes neurological recovery, peri-lesional, and contralesional neuronal plasticity. We examined effects of the HMG-CoA reductase inhibitor rosuvastatin (0.2 or 2.0 mg/kg/day i.c.v.), administered starting 3 days after 30 min of middle cerebral artery occlusion for 30 days. Here, we show that rosuvastatin treatment significantly increased the grip strength and motor coordination of animals, promoted exploration behavior, and reduced anxiety. It was associated with structural remodeling of peri-lesional brain tissue, reflected by increased neuronal survival, enhanced capillary density, and reduced striatal and corpus callosum atrophy. Increased sprouting of contralesional pyramidal tract fibers crossing the midline in order to innervate the ipsilesional red nucleus was noticed in rosuvastatin compared with vehicle-treated mice, as shown by anterograde tract tracing experiments. Western blot analysis revealed that the abundance of HMG-CoA reductase was increased in the contralesional hemisphere at 14 and 28 days post-ischemia. Our data support the idea that HMG-CoA reductase inhibition promotes brain remodeling and plasticity far beyond the acute stroke phase, resulting in neurological recovery.

## Introduction

Major efforts have been made in recent years to promote stroke recovery by stimulation of axonal sprouting (Hermann and Chopp, 2012). A variety of strategies have been used for this purpose. Antibodies aiming at the neutralization of axonal growth inhibitors [e.g., Nogo-A (Papadopoulos et al., 2002; Wiessner et al., 2003)], pleiotropic growth factors [e.g., erythropoietin, vascular endothelial growth factor (Reitmeir et al., 2011, 2012)], and neural precursor/stem cells (Bacigaluppi et al., 2009; Andres et al., 2011) have been administered. These treatments are not easily transferrable to human patients due to the inexistence of systemic delivery strategies and/or potential side effects and complications that endanger therapeutic success [e.g., brain inflammation in case of antibodies targeting CNS epitopes (Orgogozo et al., 2003) or malignant transformation in case of cell-based therapies (Amariglio et al., 2009)]. Hence, the clinical translation of plasticity-promoting therapies is still on the way.

Following the stroke prevention by aggressive reduction in cholesterol levels trial (Amarenco et al., 2006), 3-hydroxy-3-methylglutaryl-coenzyme A (HMG-CoA) reductase inhibitors (also called statins) are widely used for secondary stroke prevention. Besides their cholesterol-lowering properties, HMG-CoA reductase inhibitors exert pleiotropic effects in the brain that are beneficial for stroke recovery, promoting post-ischemic neuronal survival (Sironi et al., 2003; Kilic et al., 2005), inhibiting inflammatory responses (Pahan et al., 1997; Kilic et al., 2005), restoring endothelial function (Endres et al., 1998; Amin-Hanjani et al., 2001), and promoting angiogenesis and neurogenesis (Chen et al., 2003).

Based on these multiple observations, we now examined if HMG-CoA reductase inhibition influences neurological recovery and brain plasticity in the post-acute stroke phase. Thus, we exposed mice to intraluminal middle cerebral artery occlusion (MCAO) and investigated effects of the HMG-CoA reductase inhibitor rosuvastatin, administered starting 3 days post-ischemia (dpi), on functional neurological recovery, peri-lesional brain remodeling, and contralesional pyramidal tract plasticity.

## Materials and Methods

### Experimental groups and interventions

Experiments were performed using male C57Bl6/j mice (23–25 g) in accordance to National Institutes of Health Guidelines for the Care and Use of Laboratory Animals with local government approval (Istanbul Medipol University, Turkey). A total of four sets of mice were examined:

The first set of mice was exposed to 30 min of left-sided MCAO. At 72 h post-ischemia, animals received implantations of cannula connected to miniosmotic pumps (Alzet 2004; Alzet, Cupertino, CA, USA) into the left lateral ventricle that were randomly filled with vehicle (0.9% NaCl) or rosuvastatin (0.2 or 2 mg/kg/day diluted in 0.9% NaCl) (*n* = 10 animals/group). These miniosmotic pumps were left in place during the subsequent 4 weeks and then removed. At 42 dpi, animals were sacrificed by transcardiac perfusion with 0.9% NaCl. These animals were used for functional neurological studies and conventional histochemistry (Figure S1A in Supplementary Material).

The second set of mice was subjected to 30 min MCAO, followed by implantation of miniosmotic pumps filled with vehicle or rosuvastatin (0.2 or 2 mg/kg/day) 72 h later using the same protocol (*n* = 10 animals/group). The miniosmotic pumps were again left in place for 4 weeks and then removed. At 42 dpi, animals were sacrificed by transcardiac perfusion with 4% paraformaldehyde. These animals were used for functional neurological studies, computer-based stereology, and volumetry (Figure S1B in Supplementary Material).

The third set of mice was submitted to 30 min of MCAO or sham-surgery, following by implantation of miniosmotic pumps filled with vehicle or rosuvastatin (2 mg/kg/day) 72 h later using the same protocol (*n* = 10 animals/group). The miniosmotic pumps were again left in place for 4 weeks and then removed. The animals were used for functional neurological studies and anterograde tract tracing. For this purpose, the anterograde tract tracers cascade-blue-labeled dextran amine [cascade blue (CB)] or biotinylated dextran amine (BDA) (both 10,000 MW; Molecular Probes, Eugene, OR, USA) were injected into the ipsilesional or contralesional motor cortices, respectively, at 42 dpi or 42 days post-sham surgery (Reitmeir et al., 2011, 2012). Ten days later (i.e., at 52 dpi), animals were sacrificed by transcardiac perfusion with 4% paraformaldehyde (Figure S1C in Supplementary Material).

The last set of mice was exposed to 30 min of MCAO followed by 3, 14, 28, or 42 days reperfusion or to sham-surgery followed by 3 days reperfusion (*n* = 4 animals/group) (Kilic et al., 2008). Animals were sacrificed by transcardiac perfusion with 0.9% NaCl. Brains were used for Western blot analysis of HMG-CoA reductase abundance (Figure S1D in Supplementary Material).

Animals were always randomly attributed to experimental groups in a blinded manner. Experimenters analyzing the data were blinded for experimental conditions.

### Induction of focal cerebral ischemia

Animals were anesthetized with 1% isoflurane (30% O_2_, remainder N_2_O). Rectal temperature was maintained between 36.5 and 37.0°C using a feedback-controlled heating system. During the experiments, cerebral blood flow was measured by laser Doppler flow (LDF) recordings using a flexible 0.5 mm fiberoptic probe (Perimed, Stockholm, Sweden), which was attached to the intact skull overlying the middle cerebral artery territory (2 mm posterior/6 mm lateral from bregma). LDF changes were monitored up to 30 min after the onset of reperfusion. For intraluminal MCAO, a midline neck incision was made, and the left common and external carotid arteries were isolated and ligated. A microvascular clip (FE691; Aesculap, Tuttlingen, Germany) was temporarily placed on the internal carotid artery. A 8-0 nylon monofilament (Ethilon; Ethicon, Norderstedt, Germany) coated with silicon resin (Xantopren; Bayer Dental, Osaka, Japan; diameter of the coated filament: 180–190 μm) was introduced through a small incision into the common carotid artery and advanced 9 mm distal to the carotid bifurcation for MCAO. Thirty minutes later, reperfusion was initiated by filament removal. In sham-operated animals, a surgical intervention was performed, in which the neck was opened and the common carotid artery was exposed, but left intact, while LDF recordings were performed. After the surgery, wounds were carefully sutured, anesthesia was discontinued and animals were placed back into their cages.

Animals belonging to animal sets one to three (see Experimental Groups and Interventions) were reanesthetized with 1% isoflurane at 72 h post-ischemia. Cannula (Brain infusion kit 3; Alzet, Cupertino, CA, USA) were implanted into the left lateral ventricle (0.0 mm from bregma, 0.8 mm lateral to midline, 1.4 mm below brain surface), which were linked to miniosmotic pumps (Alzet 2004; Alzet), which were randomly filled with vehicle (0.9% NaCl) or rosuvastatin (sc-208316; Santa Cruz, Heidelberg, Germany; 0.2 or 2 mg/kg/day diluted in 0.9% NaCl) and which were placed on the animals backs. These pumps were left in place during the subsequent 4 weeks and then removed.

Animals belonging to animal set three (see Experimental Groups and Interventions) were reanesthetized with 1% isoflurane at 42 dpi or 42 days post-sham surgery. The anterograde tract tracers CB and BDA [both 10,000 MW; 10% dilutions in 0.01 M phosphate-buffered saline (PBS) at pH 7.2; Molecular Probes, Eugene, OR, USA] were injected into the ipsilesional or contralesional motor cortices, respectively, by means of microsyringe injections, as previously reported (Reitmeir et al., 2011, 2012). As such, a total volume of 2.1 μl tracer was administered to each animal, which was injected in three equal deposits located rostrally, medially, and caudally of the needle insertion site into the motor cortex. For this purpose, the needle was inclined 45°, 90°, and 135° against the midline and 45° against the brain surface (needle insertion for all three deposits: 0.5 mm rostral to bregma/2.5 mm lateral to midline; injection ~0.8 mm below brain surface) (Reitmeir et al., 2012, 2011).

### Functional neurological tests

Neurological recovery was assessed using grip strength, RotaRod, open field, elevated O maze, and light/dark tests at baseline and on days 7, 14, 28, and 42 after MCAO (Kilic et al., 2008, 2010; Reitmeir et al., 2011).

#### Grip strength test

The grip strength test consists of a spring balance coupled with a Newtonmeter (Medio-Line Spring Scale, metric, 300 g, Pesola, Switzerland) that is attached to a triangular steel wire, which the animal instinctively grasps. When pulled by the tail, the animal exerts force on the steel wire (Kilic et al., 2010). Grip strength was evaluated at the right paretic forepaw, the left non-paretic forepaw being wrapped with adhesive tape. Grip strength was evaluated five times on occasion of each test, for which mean values were calculated. From these data, percentage values (post-ischemic vs. pre-ischemic) were computed. Pre-ischemic results did not differ between groups.

#### RotaRod test

The RotaRod is a rotating drum with a speed accelerating from 6 to 40 rpm (model 47600; Ugo Basile, Comerio, Italy), which allows to assess motor coordination skills (Kilic et al., 2010). Maximum speed is reached after 245 s, and the time at which the animal drops off the drum is evaluated (maximum testing time: 300 s). Measurements were performed five times each on the same occasion when grip strength was evaluated. For all five measurements, mean values were computed, from which percentage values (post-ischemic vs. pre-ischemic) were calculated. Pre-ischemic data did not differ between groups.

#### Open field test

The open field is a round arena (diameter: 150 cm) covered by a white plastic floor, surrounded by a 35 cm high sidewall made of white polypropylene, which allows to measure spontaneous locomotor activity and exploration behavior (Kilic et al., 2008). The arena is divided into three sections, including an outer wall zone (17.7% of diameter, close to the wall), an intermediate transition zone (32.3% of diameter), and an inner zone (50% of diameter, the center of the arena). Each mouse was released near the wall and observed for 10 min. Animal paths were tracked with an electronic imaging system (Ethovision XT6; Noldus Information Technology, Wageningen, Netherlands), acquiring data at a frequency of 4.2 Hz with a spatial resolution of 576 × 768 pixels. Raw data were transferred to the wintrack 2.4 software for offline analysis. To determine measures of exploratory behavior and anxiety, the time resting and progressing, and the time spent in each of the three zones were assessed.

#### Elevated O maze

The elevated O maze consists of a round 5.5 cm wide polyvinyl-chloride runway with an outer diameter of 46 cm, which is placed 40 cm above the floor and measures correlates of fear and anxiety (Reitmeir et al., 2011). Two opposing 90° sectors are protected by 16 cm high inner and outer walls made of polyvinyl-chloride (closed sectors). The remaining two 90° sectors are not protected by walls (open sectors). Each mouse was released in one of the open sectors and observed for 10 min. Animal paths were again tracked with an electronic imaging system (Ethovision XT6). The time spent in the unprotected sector was measured whenever the animal entered this sector with all four paws.

#### Light/dark transition test

The light/dark transition test (40 cm × 20 cm × 20 cm) consists of two equal light and dark chambers, which are separated by a divider with a 4 cm × 4 cm opening at the floor level. Each mouse was placed in the corner of the light chamber at distance from the dark chamber and monitored for 10 min. Animal paths were tracked with an electronic imaging system (Ethovision XT6). The time spent in the light chamber was evaluated and presented.

### Immunohistochemical analysis of brain remodeling

#### Conventional immunohistochemistry

For conventional immunohistochemistry, 20 μm coronal sections were obtained at the level of the bregma (i.e., midstriatum) from animals that had been transcardially perfused with 0.9% NaCl at 42 dpi. After immersion fixation in 4% paraformaldehyde in 0.1 M PBS, sections were pre-treated for antigen retrieval with 0.01 M citrate buffer (pH 5.0), rinsed and immersed for 1 h in 0.1 M PBS containing 0.3% Triton X-100 (PBS-T) and 10% normal donkey serum. Sections were incubated overnight at 4°C with monoclonal mouse anti-NeuN (MAB377; Chemicon) and monoclonal rat anti-CD31 (#557355; BD Biosciences) antibodies (diluted 1:100 in 0.1 M PBS) that were detected with Cy3- or Cy2-conjugated secondary antibodies. Sections were finally incubated with 4′-6-diamidino-2-phenylindole (DAPI). In some experiments, primary antibodies were recognized by biotinylated secondary antibodies that were detected using an avidin–biotin kit (Vector Laboratories, Burlingame, CA, USA) by 3,3′-diaminobenzidine (DAB) staining. Sections were evaluated under a fluorescence microscope (Olympus BX41) connected to a CCD camera (CC12; Olympus). Surviving NeuN+ neurons and CD31+ microvessels were analyzed in a blinded way by counting numbers of cells or profiles in six defined regions of interests (ROI) in each striatum both ipsilateral and contralateral to the stroke (250 μm × 250 μm) (Kilic et al., 2006), for which mean values were calculated. With these measurements, neuronal survival and capillary density were determined. Stereometric analysis of post-ischemic striatum and corpus callosum atrophy was done using brain sections stained with modified Bielschowsky’s silver solution, as previously described (Reitmeir et al., 2011).

#### Computer-based stereological analysis and brain volumetry

For stereological analysis of brain remodeling and brain volumetry, 20 μm coronal sections were obtained from six equidistant brain levels, 250 μm apart, of animals that had been transcardially perfused with 4% paraformaldehyde in 0.1 M PBS at 42 dpi. Sections were pre-treated for antigen retrieval with 0.01 M citrate buffer (pH 5.0), rinsed and immersed for 1 h in 0.1 M PBS-T and 10% normal donkey serum. Sections were incubated overnight at 4°C with Alexa Fluor 488-conjugated monoclonal mouse anti-NeuN (Mab377X; Chemicon), polyclonal rabbit anti-CD31 (ab28364; Abcam), and Alexa Fluor 555-conjugated monoclonal mouse anti-GFAP (#3656; Cell Signaling) antibodies (diluted 1:100 in 0.1 PBS) that – in case of the non-conjugated antibody – was detected with Alexa Fluor 488-conjugated secondary antibody (A21206; Invitrogen). Sections were finally incubated with DAPI.

Sections were analyzed using a confocal Zeiss LSM 780 microscope. A software-controlled motorized stage provided accurate and fine movements for the *x*-, *y*-, and *z*-axes. In order not to miss any cell nuclei, the focus was adjusted from top to bottom for each slice. When the first nuclei were detected in the focus, the *z*-axis was determined as top point and when last nuclei were lost in the focus, the *z*-axis was determined as bottom point. For each slice, the distance between the top and bottom was ~16 μm, which was divided into four focal planes and all images were further processed as a multiple intensity projection using Zen Black software to obtain sharp images. Using tile and *z*-stack functions of the motorized stage, all signals from NeuN+, CD31+, and GFAP+ cells in the striatum were detected using the GaAsP detector of the microscope. A single investigator analyzed all data in a blinded manner. Mean numbers of NeuN+ cells were analyzed in the ischemic and contralesional striatum. By dividing results obtained in both hemispheres, the percentage of surviving neurons in the ischemic striatum was determined. CD31+ microvessels were counted in the entire ipsilateral striatum. With the data obtained, the mean capillary number was calculated. In case of GFAP stainings, the area of scar tissue was outlined using the Zen Blue software (version 2012; Carl Zeiss). With all areas from various brain levels, the scar volume in cubic millimeter was calculated. For brain volumetry, sections collected throughout the forebrain at 250 μm intervals were stained with cresyl violet. On each section, the ipsilesional striatum and ipsilesional corpus callosum (that included the external capsule) were outlined using the Zen Blue software. By integrating areas measured across the brain, striatum, and corpus callosum volumes in cubic millimeter were determined.

### Immunohistochemistry for CB and BDA

Brain sections of animals that had transcardially been perfused with paraformaldehyde were rinsed three times for 10 min each in 50 mM Tris-buffered saline (pH 8.0) containing 0.5% Triton X-100 (TBS-T). For detection of CB, sections were immersed overnight at 4°C with polyclonal rabbit anti-cascade blue antibody (A-5760; Molecular Probes, 1:100), diluted 1:100 in 50 mM TBS-T, followed by incubation for 1 h at room temperature with a horseradish peroxidase-labeled secondary antibody (1:1000). For detection of BDA, sections were incubated overnight with avidin–biotin–peroxidase complex (ABC Elite; Vector Laboratories), followed by DAB staining.

### Analysis of corticorubral projections

To account for variabilities in tracer uptake in different mice, we first evaluated the number of tracer-stained fibers in the pyramidal tract at the level of the parvocellular red nucleus (bregma −3.0 to −3.5 mm). For this purpose, two consecutive sections were analyzed, counting the number of fibers crossing the sections in four regions of interest of 2865 μm^2^ each that had been selected in the dorsolateral, ventrolateral, dorsomedial, and ventromedial portion of the pyramidal tract. By measuring the total area of the pyramidal tract using the Cell Software image system (Olympus) connected to an Olympus BX42 microscope, we calculated the overall number of labeled pyramidal tract fibers (Z’Graggen et al., 1998; Reitmeir et al., 2011).

For evaluation of midline-crossing fibers, a 500 μm long-intersection line was superimposed on the brain midline. Along that line those fibers crossing into the contralateral hemisphere in direction of the red nucleus were quantified. For each animal and both tracers, the total number of fibers counted was normalized with the total number of labeled fibers in the pyramidal tract, as determined for each tracer. This value was multiplied by 100, resulting in percent values of fibers crossing the midline. For both tracers always two consecutive sections were analyzed, of which mean values were determined.

### Western blotting

Tissue samples were collected from both middle cerebral artery territories of animals sacrificed by transcardiac perfusion with 0.9% NaCl (Reitmeir et al., 2011). Following sodium dodecyl sulphate–polyacrylamide gel electrophoresis, polyvinylidene fluoride membranes were incubated with rabbit polyclonal HMG-CoA reductase antibody (Ab98018; Abcam, Cambridge, UK) that was detected by chemiluminescence labeling. Protein loading was controlled using a β-actin antibody. Protein abundance was evaluated by densitometry. Three independent blots were analyzed. For these blots, mean values were calculated, which were normalized with optical densities determined in corresponding samples of sham-operated mice.

### Statistical analysis

Neurological tests were evaluated by means of two-way repeated measurement analysis of variance (ANOVA) for all four time-points starting at 7 days post-stroke, i.e., the first examination after MCAO had been induced. For those tests, in which significant treatment or treatment by time interaction effects were noticed, *post hoc* comparisons were performed for each time-point using unpaired *t*-tests with Bonferroni corrections. Western blotting and histochemical data were evaluated by one-way ANOVA followed by least significant differences tests (comparison between ≥3 groups) or unpaired *t*-tests (comparisons between 2 groups). *p*-Values <0.05 were considered significant.

## Results

### Post-acute delivery of rosuvastatin improves post-ischemic neurological recovery

**Figure 1:**
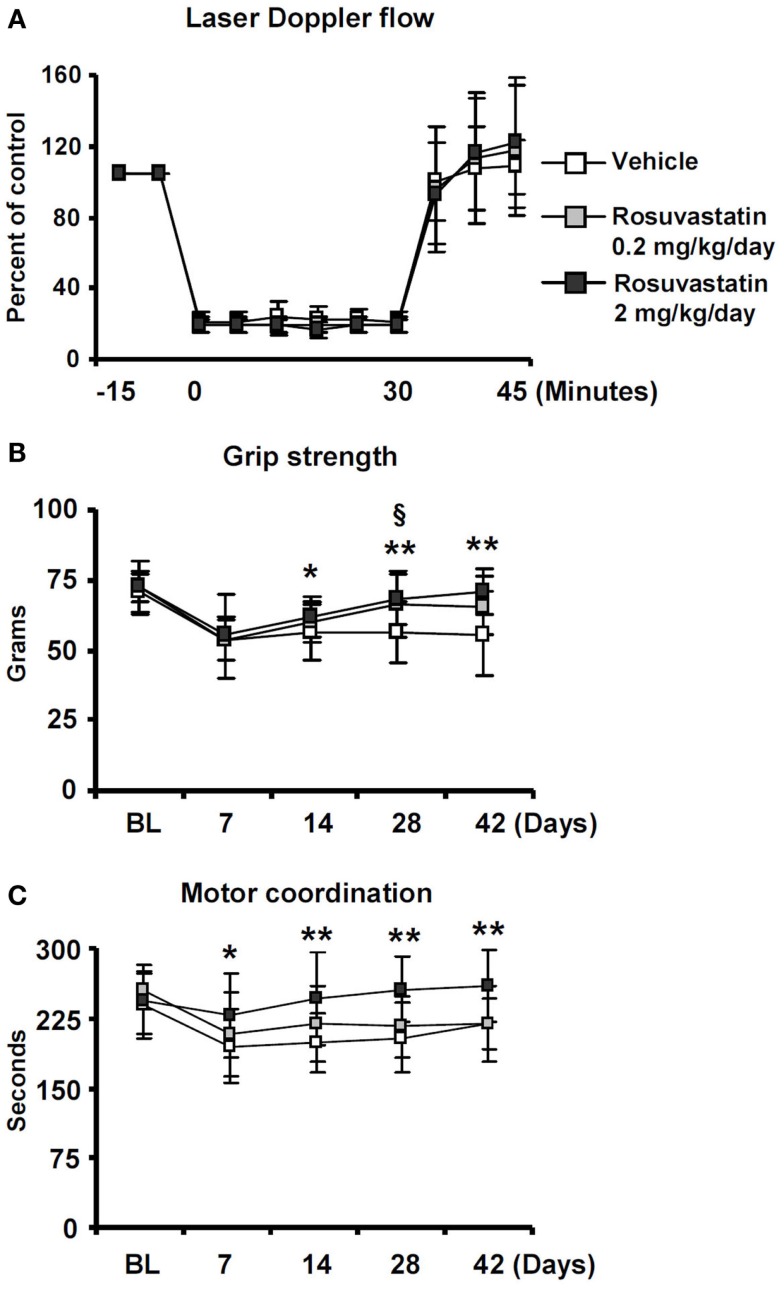
**Delayed delivery of rosuvastatin at a dose of 2 mg/kg/day, but not 0.2 mg/kg/day, promotes post-ischemic recovery of grip strength and motor coordination**. **(A)** Laser Doppler flow (LDF) recordings above the core of the middle cerebral artery territory, **(B)** grip strength of the lesion-contralateral right paretic forepaw, and **(C)** motor coordination evaluated by RotaRod tests of ischemic mice treated with vehicle or rosuvastatin (0.2 or 2 mg/kg/day i.c.v.) starting at 72 h post-ischemia. Note that motor force **(B)** and coordination skills **(C)**, which are compromised following stroke, do not exhibit any major improvements over time in vehicle-treated mice and mice receiving rosuvastatin at a dosage of 0.2 mg/kg/day, but progressively improve over 14–42 days in animals treated with rosuvastatin at a 2 mg/kg/day dosage. LDF measurements **(A)**, which were recorded for evaluating the reproducibility of ischemia, do not differ between groups. BL, baseline. Data are mean values ± SD (*n* = 20 animals/group). **p < 0.01/*p < 0.05 for high-dose rosuvastatin (2.0 mg/kg/day) compared with ischemic vehicle; ^§^*p* < 0.05 for low-dose rosuvastatin (0.2 mg/kg/day) compared with ischemic vehicle.

To evaluate if the HMG-CoA reductase inhibitor rosuvastatin influences neurological recovery, mice submitted to 30 min of MCAO were intraventricularly treated with vehicle or rosuvastastin (2 or 0.2 mg/kg/day) starting at 3 dpi, i.e., at a time-point, at which acute ischemic injury has already evolved (Reitmeir et al., 2011, 2012). LDF, which was recorded above the core of the middle cerebral artery territory using a flexible fiberoptic probe that was attached to the animals’ skulls, did not show any differences between groups. In all experimental conditions, LDF decreased to ~15–20% of baseline during MCAO, followed by a rapid restoration of blood flow after reperfusion (Figure 1A).

Significant reductions of motor force of the contralesional right forepaw (Figure 1B) and motor coordination skills (Figure 1C) were detected in ischemic mice, as shown in grip strength and RotaRod tests. In vehicle-treated mice and in mice receiving rosuvastatin at the low dosage (0.2 mg/kg/day), grip strength, and coordination skills largely remained unchanged over the observation period of 42 days (Figures 1B,C). In animals treated with rosuvastatin at the higher dosage (2 mg/kg/day), progressive recovery of grip strength and motor coordination were observed, resulting in robust and significant improvement starting at 14 dpi (Figures 1B,C).

**Figure 2:**
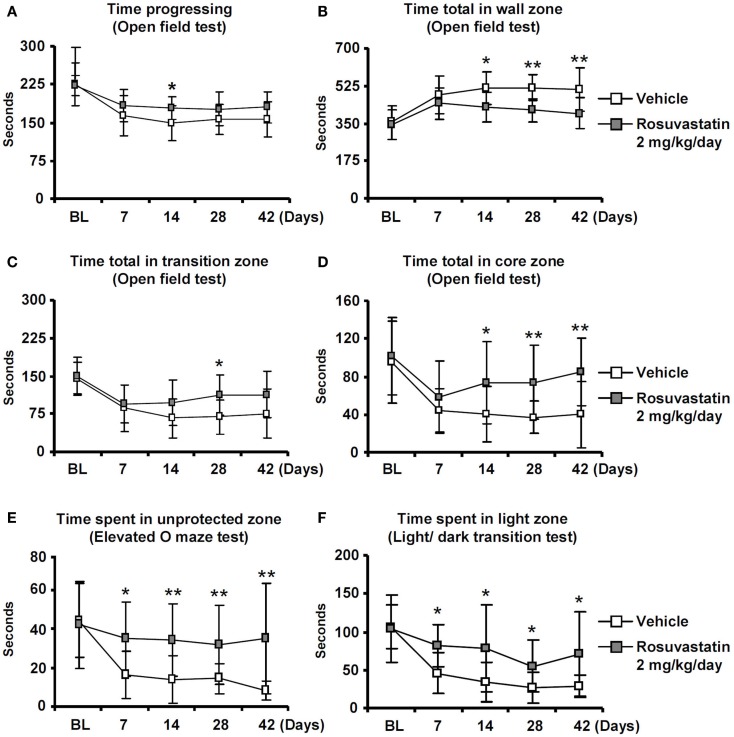
**Delayed delivery of rosuvastatin promotes post-ischemic spontaneous exploration behavior, thus, reducing anxiety**. **(A)** Time progressing, **(B)** time spent in the wall zone, **(C)** time spent in the transition zone, and **(D)** time spent in the core zone of the open field test, as well as **(E)** time spent in the unprotected zone of the elevated O maze and **(F)** time spent in the light zone of the light/dark transition test of ischemic mice treated with vehicle or rosuvastatin (2 mg/kg/day i.c.v.) starting at 72 h post-ischemia. Note that while overall motor activity does not differ between groups **(A)**, exploration activities of ischemic mice in the transition zone **(C)**, and core zone **(D)** of the open field are increased by rosuvastatin. Furthermore, note that the time spent in the unprotected zone of the elevated O maze **(E)** and the time spent in the light zone of the light/dark test **(F)** are increased by rosuvastatin, indicating that post-stroke anxiety is attenuated by HMG-CoA reductase inhibition BL, baseline. Data are mean values ± SD (*n* = 20 animals/group). **p < 0.01/*p < 0.05 compared with ischemic vehicle.

Whereas overall locomotor activity that was reduced by MCAO was mildly increased by rosuvastatin, as shown in open field tests (Figure 2A), rosuvastatin decreased the time spent in the open field wall zone (Figure 2B) and increased the time spent in the open field transition zone (Figure 2C) and center zone (Figure 2D), indicating partial reversal of an anxious phenotype that was induced by the stroke. In line with this promotion of exploration behavior, the time spent in the unprotected zone of the elevated O maze (Figure 2E) and the time spent in the light zone of the light/dark transition test (Figure 2F) were significantly increased by rosuvastatin at a 2 mg/kg/day dosage. In view that effects on grip strength and coordination skills were noticed only when the higher dose of rosuvastatin was administered, the latter studies and all following studies were performed only in mice receiving vehicle or 2 mg/kg/day rosuvastatin.

### Rosuvastatin promotes peri-lesional tissue remodeling

**Figure 3:**
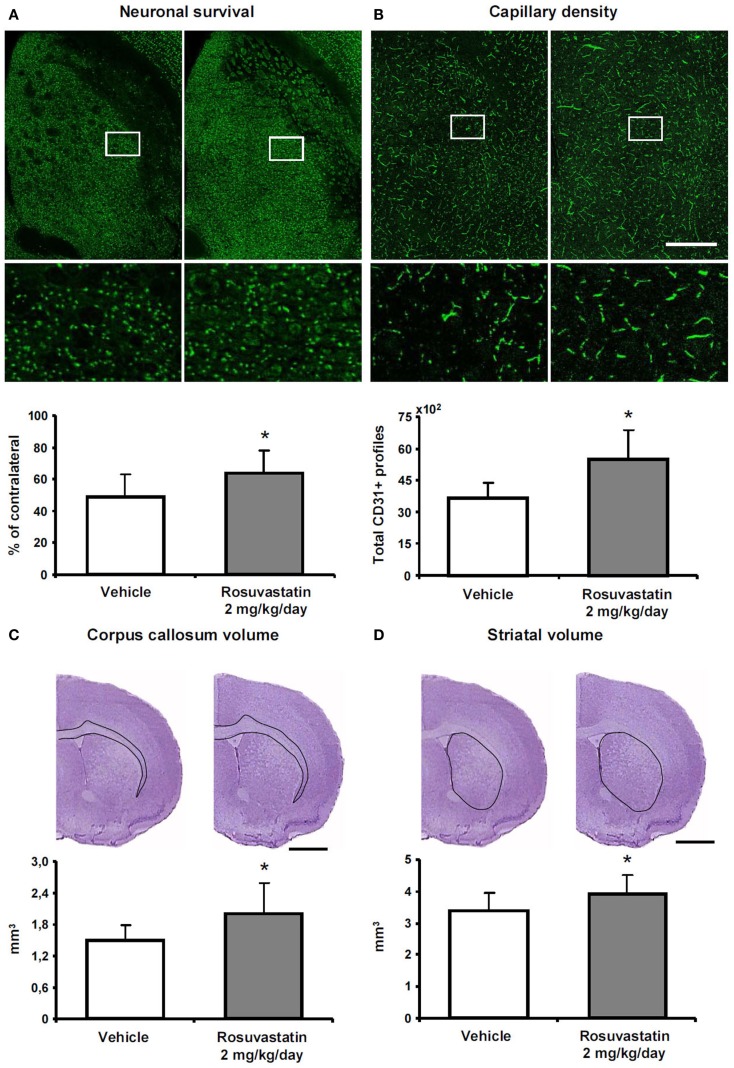
**Rosuvastatin promotes peri-lesional tissue remodeling**. **(A)** Surviving neurons in ischemic striatum evaluated by NeuN immunohistochemistry, **(B)** striatum atrophy, and **(C)** corpus callosum atrophy examined by cresyl violet stainings, and **(D)** capillary number in the ischemic striatum assessed by CD31 immunohistochemistry at 42 dpi in ischemic mice receiving vehicle or rosuvastatin (2 mg/kg/day i.c.v.) starting at 72 h post-ischemia. Data were analyzed using an investigator-independent computer-based stereology and volumetry approach. Note that rosuvastatin increases neuronal survival **(A)**, diminishes progressive brain atrophy **(B,C)**, and increases capillary survival **(D)**. Representative microphotographs are also shown. Data are mean values ± SD (*n* = 10 animals/group). *p < 0.05 compared with ischemic vehicle. Bars, 1000 μm **(A–D)**.

To clarify how the post-acute rosuvastatin delivery modifies the remodeling of ischemic brain tissue, we used two different approaches, i.e., conventional histochemical analysis and computer-based stereology and volumetry, always with consistent results in two different sets of mice. Rosuvastatin at a dose of 2 mg/kg/day significantly increased neuronal survival after the observation period of 42 days (Figure 3A; Figure S2A in Supplementary Material), preventing striatum (Figure 3B; Figure S2B in Supplementary Material) and corpus callosum (Figure 3C; Figure S2C in Supplementary Material) atrophy. Brain capillary density was increased in the lesion-sided striatum of rosuvastatin-treated as compared with vehicle-treated ischemic mice (Figure 3D; Figure S2D in Supplementary Material), but not the overlying parietal cortex (not shown). Glial scar formation was slightly reduced by rosuvastatin (1.76 ± 0.64 vs. 1.44 ± 0.60 mm^3^ in vehicle-treated compared with rosuvastatin-treated animals); yet, this effect failed significance.

### Rosuvastatin promotes contralesional corticorubral tract plasticity

**Figure 4:**
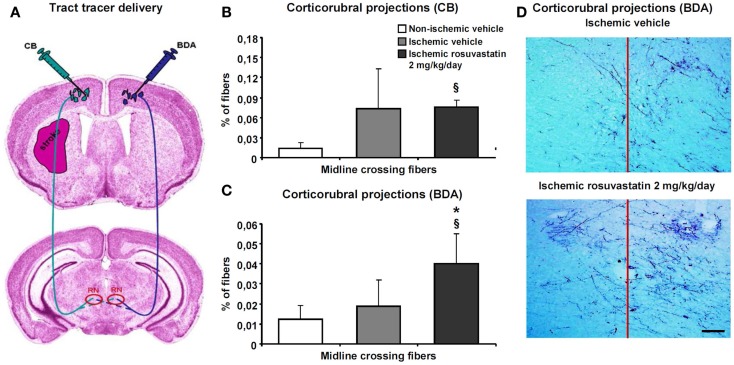
**Rosuvastatin promotes contralesional corticorubral plasticity**. Tract tracing analysis of corticorubral projections ipsilateral and contralateral to the stroke in mice receiving cascade blue (CB) and biotinylated dextran amine (BDA) injections into the lesion-sided and contralesional motor cortex [for experimental procedures see **(A)**]. Percent of midline-crossing fibers to **(B)** the contralesional red nucleus (RN) traced by CB and **(C)** ipsilesional red nucleus traced by BDA. Note that the percentage of midline-crossing fibers after ipsilesional CB injection increases in response to stroke (though not significantly) **(B)**. Importantly, rosuvastatin, delivered at a dose of 2 mg/kg/day, does not further elevate the percentage of midline-crossing fibers originating from the ipsilesional pyramidal tract **(B)**, but increases the percentage of contralesional pyramidal tract fibers growing out across the midline in direction to the ipsilesional red nucleus **(C)**. **(D)** Microphotographs of representative vehicle-treated and rosuvastatin-treated ischemic mice illustrating BDA traced corticorubral fibers crossing the midline in between both red nuclei. Note that the ipsilesional (left) red nucleus receives more BDA traced fibers after rosuvastatin than vehicle delivery. Data are mean values ± SD (*n* = 10 animals/group). ^§^*p* < 0.05 compared with non-ischemic vehicle; *p < 0.05 compared with ischemic vehicle. Bar, 100 μm.

Since the pyramidal tract crosses the middle cerebral artery territory, which was affected by ischemia, we examined how rosuvastatin influences pyramidal tract degeneration and plasticity. We administered two dextran conjugates, CB and BDA, into both motor cortices (Figure 4A). The analysis of injection sites revealed no relevant differences between groups. In all animals, injection sites covered the more caudal forelimb area and rostral hindlimb area of the primary motor cortex without relevant spreading of tracer deposits into subcortical structures. The size of the pyramidal tract, evaluated as area covered by the cerebral peduncle in coronal brain sections, was slightly lower in the ipsilesional ischemic, as compared to non-ischemic pyramidal tract, but was not influenced by rosuvastatin (10.8 ± 3.5 mm^2^ in non-ischemic vehicle, 8.0 ± 1.8 mm^2^ in ischemic vehicle, 8.0 ± 2.3 mm^2^ in ischemic rosuvastatin; *p* < 0.05 for ischemic rosuvastatin and ischemic vehicle compared with non-ischemic vehicle).

Cascade blue- and BDA-stained fibers originating from the cerebral peduncle turned dorsomedially at mesencephalic levels, terminating as previously described in the parvocellular part of the ipsilateral red nucleus (Figure 4A) (Reitmeir et al., 2011). At this level, we quantified both the number of fibers crossing through the pyramidal tract and the number of fibers branching off the pyramidal tract and migrating across the midline in direction to the red nucleus in the other hemisphere. Our studies revealed that MCAO increased the number of CB-labeled fibers in the ipsilesional cerebral peduncle, whereas rosuvastatin delivery did not further elevate this number (19171 ± 6026 CB-labeled fibers in non-ischemic vehicle, 35942 ± 19114 CB-labeled fibers in ischemic vehicle, 38425 ± 11567 CB-labeled fibers in ischemic rosuvastatin; *p* < 0.01 for ischemic rosuvastatin and ischemic vehicle compared with non-ischemic vehicle). Conversely, neither MCAO nor rosuvastatin influenced the number of labeled fibers in the contralesional cerebral peduncle (48300 ± 19429 BDA-labeled fibers in non-ischemic vehicle, 55944 ± 17875 BDA-labeled fibers in ischemic vehicle, 55988 ± 14028 BDA-labeled fibers in ischemic rosuvastatin; n.s.). Hence, rosuvastatin did not induce the *de novo* formation of proximal axons, neither ipsilateral nor contralateral to the stroke.

The percentage of midline-crossing fibers derived from the lesion-sided pyramidal tract, as revealed by CB, increased (though not significantly) upon MCAO (Figures 4B,D), whereas the percentage of BDA-labeled midline-crossing fibers originating from the contralesional pyramidal tract remained unchanged (Figures 4C,D). Importantly, rosuvastatin significantly increased the percentage of midline-crossing fibers originating from the BDA-labeled contralesional pyramidal tract, without influencing the percentage of CB-labeled ipsilesional pyramidal tract fibers (Figures 4B–D). Thus, rosuvastatin enhanced the sprouting of terminal fibers originating from the contralateral, but not ipsilateral motor cortex.

### HMG-CoA reductase is upregulated in the contralesional hemisphere

**Figure 5:**
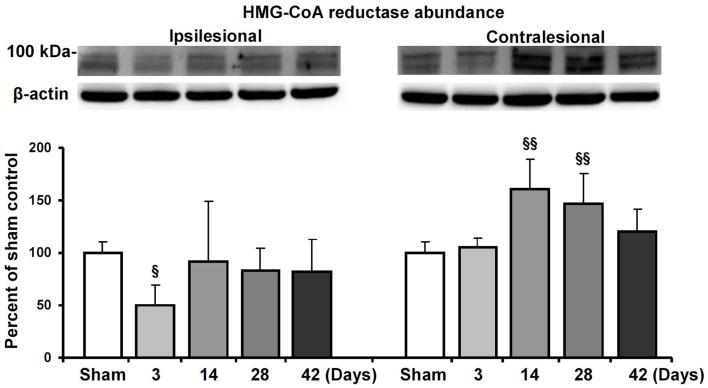
**HMG-CoA reductase abundance in the ischemic and contralesional hemisphere**. Western blot analysis of the ~100 kDa double band of HMG-CoA reductase at various reperfusions times following 30 min MCAO. Representative blots are also shown. Note the upregulation of HMG-CoA reductase in the contralesional hemisphere at 14 and 28 d.p.i. Data are mean values ± SD (*n* = 3 independent blots/group). ^§§^*p* < 0.01/^§^*p* < 0.05 compared with corresponding samples from sham-operated mice.

Based on the observation that HMG-CoA reductase inhibition enhanced contralesional pyramidal tract plasticity, we finally analyzed how focal cerebral ischemia influences HMG-CoA reductase abundance both ipsilateral and contralateral to the stroke. In Western blots, we show that the abundance of HMG-CoA reductase increased in the contralateral hemisphere at 14 and 28 dpi in mice exposed to 30 min MCAO (Figure 5). In the ipsilesional hemisphere, HMG-CoA reductase abundance transiently decreased at 3 dpi.

## Discussion

We herein show that post-acute delivery of the HMG-CoA reductase inhibitor rosuvastatin, initiated at 72 h post-ischemia, that is a time-point far beyond classical neuroprotection, promotes neurological recovery, peri-lesional tissue remodeling, and contralesional pyramidal tract plasticity in mice submitted to MCAO. Robust correlates of recovery of motor function, coordination skills, and exporation behavior were noticed in functional neurological tests that were accompanied by decreased neuronal degeneration and brain atrophy, and increased capillary survival. Contralateral to the stroke, enhanced sprouting of midline-crossing motor cortical fibers was induced by rosuvastatin, providing another structural correlate for the improved neurological recovery.

The observation of increased axonal plasticity in response to HMG-CoA reductase inhibitor delivery that occurs at distance to the lesion is new. It has previously been shown that HMG-CoA reductase inhibition promotes post-ischemic neuronal survival in the acute stroke phase (Sironi et al., 2003; Kilic et al., 2005), inhibit brain inflammation (Pahan et al., 1997; Kilic et al., 2005), restore endothelial function (Endres et al., 1998; Amin-Hanjani et al., 2001), and promote angiogenesis and neurogenesis (Chen et al., 2003). Following exposure of primary cortical neurons that had undergone oxygen–glucose deprivation to the HMG-CoA reductase inhibitor simvastatin, increased neurite outgrowth has been reported, which was made responsible for the increased remodeling of Bielschowsky-labeled fiber tracts along the lesion border that was found by the same authors in simvastatin treated rats subjected to 2 h MCAO (Cui et al., 2012). These observations in the vicinity of the stroke lesion are in line with our findings of reduced striatum and corpus callosum atrophy. That corpus callosum atrophy is reduced by HMG-CoA reductase inhibition has to the best of our knowledge not yet been shown. Mechanistically, neuronal plasticity induced by HMG-CoA reductase inhibitors is thought to be mediated by prevention of geranylgeranylation, as previously shown *in vitro* in rat hippocampal neurons, in which inhibition of geranylgeranylation mimicked effects of the HMG-CoA reductase inhibitor pravastatin on neuritogenesis (Pooler et al., 2006). Pravastatin significantly decreased levels of membrane-associated RhoA, suggesting that reduced geranylgeranylation of RhoA, which is required for membrane binding, was responsible (Pooler et al., 2006).

Studies in different models of MCAO using antibodies directed against axonal growth inhibitors [e.g., Nogo-A (Papadopoulos et al., 2002; Wiessner et al., 2003)], pleiotropic growth factors [e.g., erythropoietin, vascular endothelial growth factor (Reitmeir et al., 2011, 2012)], and neural precursor/stem cells (Andres et al., 2011) have in the meantime shown that contralesional pyramidal tract plasticity is a structural correlate of recovery in a variety of neurorestorative therapies. The present study shows that the HMG-CoA reductase inhibitor rosuvastatin shares this property. In contrast to antibodies, growth factors, and cell-based therapeutics, HMG-CoA reductase inhibitors may systemically be delivered without major side effects or complications. Unfortunately, HMG-CoA reductase inhibitors that are clinically used today are highly hydrophilic drugs, which poorly cross the blood–brain barrier. In order to ensure the brain access of rosuvastatin, which may still hamper in the post-ischemic brain despite blood–brain barrier opening, we used an i.c.v. delivery strategy and administered an effective drug dosage that was approximately an order of magnitude higher than that prescribed in human patients.

Since HMG-CoA reductase inhibitors are genuine pharmacological compounds that in contrast to growth factors, antibodies, or cells can easily be administered in stroke patients without concerns, more lipophilic HMG-CoA reductase inhibitors, which are able to enter the brain, might be useful neuronal plasticity-promoting drugs. Besides, HMG-CoA reductase inhibitors have very favorable effects on post-ischemic angiogenesis (Chen et al., 2003), which make them promising for restorative stroke therapy. We used a model of transient focal cerebral ischemia, in which the pyramidal tract is injured, while the motor cortex, which is located outside the middle cerebral artery territory, remains intact. Additional studies using a model, resulting in motor cortical infarcts (e.g., photothrombotic stroke) as well as studies using systemic HMG-CoA reductase inhibitor delivery may be valuable, before the translational potential of HMG-CoA reductase inhibitor-induced neuronal plasticity may finally be evaluated.

## Conflict of Interest Statement

The authors declare that the research was conducted in the absence of any commercial or financial relationships that could be construed as a potential conflict of interest.

## Supplementary Material

The Supplementary Material for this article can be found online at http://www.frontiersin.org/Journal/10.3389/fncel.2014.00422/abstract

## References

[B1] Amarenco P. Bogousslavsky J. Callahan A. III Goldstein L. B. Hennerici M. Rudolph A. E. (2006). High-dose atorvastatin after stroke or transient ischemic attack. N. Engl. J. Med. 355, 549–559. 10.1056/NEJMoa061894 16899775

[B2] Amariglio N. Hirshberg A. Scheithauer B. W. Cohen Y. Loewenthal R. Trakhtenbrot L. (2009). Donor-derived brain tumor following neural stem cell transplantation in an ataxia telangiectasia patient. PLoS Med. 6:e1000029. 10.1371/journal.pmed.1000029 19226183 PMC2642879

[B3] Amin-Hanjani S. Stagliano N. E. Yamada M. Huang P. L. Liao J. K. Moskowitz M. A. (2001). Mevastatin, an HMG-CoA reductase inhibitor, reduces stroke damage and upregulates endothelial nitric oxide synthase in mice. Stroke 32, 980–986. 10.1161/01.STR.32.4.980 11283400

[B4] Andres R. H. Horie N. Slikker W. Keren-Gill H. Zhan K. Sun G. (2011). Human neural stem cells enhance structural plasticity and axonal transport in the ischaemic brain. Brain 134, 1777–1789. 10.1093/brain/awr094 21616972 PMC3102243

[B5] Bacigaluppi M. Pluchino S. Peruzzotti-Jametti L. Kilic E. Kilic U. Salani G. (2009). Delayed post-ischaemic neuroprotection following systemic neural stem cell transplantation involves multiple mechanisms. Brain 132, 2239–2251. 10.1093/brain/awp174 19617198

[B6] Chen J. Zhang Z. G. Li Y. Wang Y. Wang L. Jiang H. (2003). Statins induce angiogenesis, neurogenesis, and synaptogenesis after stroke. Ann. Neurol. 53, 743–751. 10.1002/ana.10555 12783420

[B7] Cui X. Chopp M. Shehadah A. Zacharek A. Kuzmin-Nichols N. Sanberg C. D. (2012). Therapeutic benefit of treatment of stroke with simvastatin and human umbilical cord blood cells: neurogenesis, synaptic plasticity, and axon growth. Cell Transplant. 21, 845–856. 10.3727/096368911X627417 22405262 PMC3442771

[B8] Endres M. Laufs U. Huang Z. Nakamura T. Huang P. Moskowitz M. A. (1998). Stroke protection by 3-hydroxy-3-methylglutaryl (HMG)-CoA reductase inhibitors mediated by endothelial nitric oxide synthase. Proc. Natl. Acad. Sci. U.S.A. 95, 8880–8885. 10.1073/pnas.95.15.8880 9671773 PMC21171

[B9] Hermann D. M. Chopp M. (2012). Promoting brain remodelling and plasticity for stroke recovery: therapeutic promise and potential pitfalls of clinical translation. Lancet Neurol. 11, 369–380. 10.1016/S1474-4422(12)70039-X 22441198 PMC3964179

[B10] Kilic E. ElAli A. Kilic U. Guo Z. Ugur M. Uslu U. (2010). Role of Nogo-A in neuronal survival in the reperfused ischemic brain. J. Cereb. Blood Flow Metab. 30, 969–984. 10.1038/jcbfm.2009.268 20087369 PMC2949191

[B11] Kilic E. Kilic U. Bacigaluppi M. Guo Z. Abdallah N. B. Wolfer D. P. (2008). Delayed melatonin administration promotes neuronal survival, neurogenesis and motor recovery, and attenuates hyperactivity and anxiety after mild focal cerebral ischemia in mice. J. Pineal Res. 45, 142–148. 10.1111/j.1600-079X.2008.00568.x 18284547

[B12] Kilic E. Kilic U. Matter C. M. Lüscher T. F. Bassetti C. L. Hermann D. M. (2005). Aggravation of focal cerebral ischemia by tissue plasminogen activator is reversed by 3-hydroxy-3-methylglutaryl coenzyme A reductase inhibitor but does not depend on endothelial NO synthase. Stroke 36, 332–336. 10.1161/01.STR.0000152273.24063.f7 15625301

[B13] Kilic E. Kilic U. Wang Y. Bassetti C. L. Marti H. H. Hermann D. M. (2006). The phosphatidylinositol-3 kinase/Akt pathway mediates VEGF’s neuroprotective activity and induces blood brain barrier permeability after focal cerebral ischemia. FASEB J. 20, 1185–1187. 10.1096/fj.05-4829fje 16641198

[B14] Orgogozo J. M. Gilman S. Dartigues J. F. Laurent B. Puel M. Kirby L. C. (2003). Subacute meningoencephalitis in a subset of patients with AD after Abeta42 immunization. Neurology 61, 46–54. 10.1212/01.WNL.0000073623.84147.A8 12847155

[B15] Pahan K. Sheikh F. G. Namboodiri A. M. Singh I. (1997). Lovastatin and phenylacetate inhibit the induction of nitric oxide synthase and cytokines in rat primary astrocytes, microglia, and macrophages. J. Clin. Invest. 100, 2671–2679 10.1172/JCI119812 9389730 PMC508470

[B16] Papadopoulos C. M. Tsai S. Y. Alsbiei T. O’Brien T. E. Schwab M. E. Kartje G. L. (2002). Functional recovery and neuroanatomical plasticity following middle cerebral artery occlusion and IN-1 antibody treatment in the adult rat. Ann. Neurol. 51, 433–441. 10.1002/ana.10144 11921049

[B17] Pooler A. M. Xi S. C. Wurtman R. J. (2006). The 3-hydroxy-3-methylglutaryl co-enzyme A reductase inhibitor pravastatin enhances neurite outgrowth in hippocampal neurons. J. Neurochem. 97, 716–723. 10.1111/j.1471-4159.2006.03763.x 16573653

[B18] Reitmeir R. Kilic E. Kilic U. Bacigaluppi M. ElAli A. Salani G. (2011). Post-acute delivery of erythropoietin induces stroke recovery by promoting perilesional tissue remodelling and contralesional pyramidal tract plasticity. Brain 134, 84–99. 10.1093/brain/awq344 21186263

[B19] Reitmeir R. Kilic E. Reinboth B. S. Guo Z. ElAli A. Zechariah A. (2012). Vascular endothelial growth factor induces contralesional corticobulbar plasticity and functional neurological recovery in the ischemic brain. Acta Neuropathol. 123, 273–284. 10.1007/s00401-011-0914-z 22109109

[B20] Sironi L. Cimino M. Guerrini U. Calvio A. M. Lodetti B. Asdente M. (2003). Treatment with statins after induction of focal ischemia in rats reduces the extent of brain damage. Arterioscler. Thromb. Vasc. Biol. 23, 322–327. 10.1161/01.ATV.0000044458.23905.3B 12588778

[B21] Wiessner C. Bareyre F. M. Allegrini P. R. Mir A. K. Frentzel S. Zurini M. (2003). Anti-Nogo-A antibody infusion 24 hours after experimental stroke improved behavioral outcome and corticospinal plasticity in normotensive and spontaneously hypertensive rats. J. Cereb. Blood Flow Metab. 23, 154–165. 10.1097/00004647-200302000-00003 12571447

[B22] Z’Graggen W. J. Metz G. A. Kartje G. L. Thallmair M. Schwab M. E. (1998). Functional recovery and enhanced corticofugal plasticity after unilateral pyramidal tract lesion and blockade of myelin-associated neurite growth inhibitors in adult rats. J. Neurosci. 18, 4744–4757. 9614248 10.1523/JNEUROSCI.18-12-04744.1998 PMC6792708

