# Supplementary material for: HMG-CoA Reductase Inhibition Promotes Neurological Recovery, Peri-Lesional Tissue Remodeling, and Contralesional Pyramidal Tract Plasticity after Focal Cerebral Ischemia
Source: Front Cell Neurosci. 2014 Dec 11;8:422. doi: 10.3389/fncel.2014.00422 (PMC4263103; doi:10.3389/fncel.2014.00422)
Supplement: Supplementary file 1 [file DataSheet_1.DOCX]

***Supplementary Material***

HMG-CoA reductase inhibition promotes neurological recovery, perilesional tissue remodeling and contralesional pyramidal tract plasticity after focal cerebral ischemia

Ertugrul Kilic, PhD;^1^ Raluca Reitmeir, MD-PhD;^2^ Ülkan Kilic, PhD;^3^ Ahmet Burak Caglayan, MSc;^1^ Mustafa Caglar Beker, MSc;^1^ Taha Kelestemur, MSc;^1^ Sinem Ethemoglu, MSc;^1^ Gurkan Ozturk, MD-PhD;^1^ Dirk M. Hermann, MD.^2*^

^1^Department of Physiology, Istanbul Medipol University, Istanbul, Turkey; ^2^Department of Neurology, University Hospital Essen, Essen, Germany; ^3^Department of Medical Biology, Istanbul Medipol University, Istanbul, Turkey

**Supplementary Figure 1. Experimental procedures and animal groups.** Mice submitted to middle cerebral artery occlusion (MCAO) were treated with vehicle or rosuvastatin from days 3–30 post-ischemia. These animals were used for **(A)** conventional histochemistry, immunohistochemistry and behavior tests, **(B)** computer-based stereology and behavior tests, **(C)** tract tracing and behavior tests and **(D)** protein expression analysis by Western blotting. Numbers of animals evaluated for each group and time point of animal sacrifice are also shown. In addition to the animals shown in this scheme, additional sham-operated animals were generated as control groups in **(C)** and **(D)**, as outlined in the ‘Materials and methods’ section.

**Supplementary Figure 2. Rosuvastatin promotes peri-lesional tissue remodeling. (A)** Surviving neurons in ischemic striatum evaluated by NeuN immunohistochemistry, **(B)** striatum atrophy and **(C)** corpus callosum atrophy examined by Bielschowski stainings and **(D)** brain capillary density in the ischemic striatum assessed by CD31 immunohistochemistry at 42 dpi in ischemic mice receiving vehicle or rosuvastatin (2 mg/kg/day i.c.v.) starting at 72 hours post-ischemia. Cell densities were analysed in six defined regions of interest in the midstriatum at the level of the bregma, considering that the midstriatum is the level, at which the middle cerebral artery territory has got its maximal extension. Note again that rosuvastatin increases neuronal survival **(A)**, diminishes progressive brain atrophy **(B, C)** and promotes capillary density **(D)**. Representative microphotographs are also shown. Data are mean values±S.D. (n=10 animals/ group). *p<0.05 compared with vehicle-treated ischemic mice. Bar, 150 µm **(A-C)**/ 1000 µm **(D)**.
